# IDENTIFYING AND PREVENTING RESIDENT-TO-RESIDENT AGGRESSION IN LONG-TERM CARE: EMERGING GLOBAL PERSPECTIVES

**DOI:** 10.1093/geroni/igad104.0146

**Published:** 2023-12-21

**Authors:** E-Shien Chang, Laura Mosqueda

## Abstract

Resident-to-resident aggression (RRA) is a widespread form of mistreatment in long-term care facilities, affecting 1 in 5 residents every month. Despite its high frequency and adverse consequences for residents, staff, and family members, RRA remains under-recognized and insufficiently studied. Several reasons exist for this, including challenges in obtaining high-quality data to inform evidence-based prevention and intervention strategies. Improved understanding of RRA aligns with recent national policy imperatives to increase nursing home safety and quality of care. To accelerate knowledge-to-practice translation, this symposium highlights expertise from North America and Asia to review the latest evidence and experiences that characterize and improve RRA. Dr. E-Shien Chang will identify the role of racial and ethnic conflicts in RRA using data from the largest RRA prevalence cohort study of U.S. nursing home residents. Dr. Elsie Yan will examine resident and environment risk factors of RRA in Hong Kong, illustrating the needs for improved dementia care training among direct care workers. Dr. David Burnes will discuss barriers, successes, and lessons learned in replicating and adapting a gold standard U.S. based RRA prevalence study in the unique context of Canada. Dr. Karl Pillemer will describe a novel dissemination model to expand program reach of Improving Resident Relationships in Long Term Care (IRRL), an evidence-based RRA intervention program. Finally, Dr. Laura Mosqueda, as policy expert of long-term care services and discussant, will moderate a discussion on translating RRA research to practice and policy changes within the global contexts of long-term care. This is a collaborative symposium between the Abuse, Neglect and Exploitation of Older Persons and Research in Quality of Care Interest Groups.

